# Bacterial Biopolymer: Its Role in Pathogenesis to Effective Biomaterials

**DOI:** 10.3390/polym13081242

**Published:** 2021-04-12

**Authors:** Sreejita Ghosh, Dibyajit Lahiri, Moupriya Nag, Ankita Dey, Tanmay Sarkar, Sushil Kumar Pathak, Hisham Atan Edinur, Siddhartha Pati, Rina Rani Ray

**Affiliations:** 1 Department of Biotechnology, Maulana Abul Kalam Azad University of Technology, Haringhata 741249, India; sreejita.ghosh@gmail.com (S.G.); ankita.dey16061996@gmail.com (A.D.); 2 Department of Biotechnology, University of Engineering & Management, Kolkata 700160, India; dibyajit.lahiri@uem.edu.in (D.L.); moupriya.nag@uem.edu.in (M.N.); 3 Department of Food Technology and Bio-Chemical Engineering, Jadavpur University, Kolkata 700032, India; tanmays468@gmail.com; 4 Malda Polytechnic, West Bengal State Council of Technical Education, Government of West Bengal, Malda 732102, India; 5 Department of Bioscience and Bioinformatics, Khallikote University, Berhampur 761008, India; skpathak.su@gmail.com; 6 School of Health Sciences, Universiti Sains Malaysia, Health Campus, Kubang Kerian 16150, Kelantan, Malaysia; 7 Centre of Excellence, Khallikote University, Berhampur 761008, India; 8 Research Division, Association for Biodiversity Conservation and Research (ABC), Odisha 756001, India

**Keywords:** cell factories, bacterial biopolymers, pathogenesis, biomaterials, synthetic biology

## Abstract

Bacteria are considered as the major cell factories, which can effectively convert nitrogen and carbon sources to a wide variety of extracellular and intracellular biopolymers like polyamides, polysaccharides, polyphosphates, polyesters, proteinaceous compounds, and extracellular DNA. Bacterial biopolymers find applications in pathogenicity, and their diverse materialistic and chemical properties make them suitable to be used in medicinal industries. When these biopolymer compounds are obtained from pathogenic bacteria, they serve as important virulence factors, but when they are produced by non-pathogenic bacteria, they act as food components or biomaterials. There have been interdisciplinary studies going on to focus on the molecular mechanism of synthesis of bacterial biopolymers and identification of new targets for antimicrobial drugs, utilizing synthetic biology for designing and production of innovative biomaterials. This review sheds light on the mechanism of synthesis of bacterial biopolymers and its necessary modifications to be used as cell based micro-factories for the production of tailor-made biomaterials for high-end applications and their role in pathogenesis.

## 1. Introduction

Biopolymers are polymers being synthesized by living organisms with the help of enzymes that connects the building blocks like sugars, hydroxyl fatty acids, and amino acids to produce molecules with high molecular weight. Bacteria produce a diverse variety of biopolymers like polyamides (amino acids linked by peptide bonds), polysaccharides (sugars or sugar acids linked by glycosidic bonds), polyphosphates (inorganic phosphates connected by anhydride bonds), and polyesters (hydroxyl fatty acids connected by ester bonds). Over a long period of time, scientists have tried to understand the pathways involved in the biosynthesis of biopolymers, since they are involved in bacterial pathogenesis and persistence. These polymers can act as shielding capsules to protect the cells and storage components and form the matrix component involved in biofilm formation that causes almost 60–80% of total human infections [1]. The physicochemical properties of these biopolymers have various medicinal as well as industrial applications [2,3,4,5]. Over the recent few years, bioengineering and synthetic biological methods helped to produce new biopolymers having potential usage in medicines (like hyaluronate that acts as a biomaterial), ingredients in various food materials (Chitosan, dextran, and xanthan), and production of polyester that is used in packaging [6,7,8,9]. The designing of microbial cell factories producing biopolymers is today a significant matter of research and commercial interest.

Even though biopolymer production consumes both nutrients and chemical energy, it is supported by bacteria, since biopolymers help them grow and persist in the presence of a wide range of unfavorable conditions, such as overcoming the immune responses inside the host cells. They are involved in carrying out various other biological roles, namely protection, energy storage, and adhesion. The production of biopolymers is mainly dependent upon environmental stimuli [10]. The physicochemical properties of biopolymers are essential to maintain cellular behaviors like attachment over the abiotic or biotic surfaces, translocation, and shielding and endurance. Bacteria are known to produce extracellular polymeric substances (EPS) that entangle within themselves to provide a mesh like network encasing the bacterial cells. This EPS production is mainly essential in the formation of biofilms, which are referred to as potentially structured communities of bacterial cells [11], and these communities are considered to be one of the most persistent forms of life on Earth. EPS are the biopolymers that are synthesized by several strains of microorganisms, providing structural stability and nutrients during adhesion of the sessile microcolonies and the development of the biofilm. EPS produced by the microbial communities vary greatly in their biochemical composition, thus exhibiting varying chemical and physical properties. Some of these can be polyanionic, whereas others can be polycationic in nature [12]. The complex arrangement of structural units inside the EPS has been studied for decades. The macromolecules that are predominantly available within the EPS comprise of polysaccharides, proteins, peptidoglycans, lipids, enzymes, and extracellular DNA (eDNA). Nuclease that is present within the EPS acts as an important regulator for the formation of the biofilm [13]. The polysaccharides are a widely studied component of EPS. The analysis of various polysaccharides obtained from varied microbial species show that they vary largely in their composition and are made up of one or more different structural components with varied arrangement in EPS [14]. The commonly found polysaccharides within the EPS are highly soluble in salt solution and water. The polysaccharides forming the capsule help in adherence to the cell surface via covalent bonds with the other polymers that are present upon the surface. The extracellular polysaccharides are insoluble and cannot be separated from the cells, thereby making the determination of physical and chemical properties difficult. As the formation of biofilms serves as the main platform for the development of chronic infections [15], there are many pieces of research to understand the function of bacterial biopolymers in the formation of biofilms leading to pathogenesis. These biopolymers produced by bacterial cellular pathways and their biological roles have been considered as potential targets for the development of new antibacterial drugs.

Besides these, researches are still going on to harness the novel materialistic properties of these bacterial biopolymers like dextran [16], cellulose [17], polyesters [18], and xanthan [19] to be used in industries to produce medicines and other materials. Over the last few years, advanced molecular methodologies and genome sequencing have provided a large amount of information regarding the role of bacterial biopolymers to cause pathogenesis and also to devise bacteria to be used as cell factories producing tailor-made biomaterials. These biodegradable and renewable materials have the potential to replace the oil-based type of materials and can also be exploited to develop novel high-end biomaterials in order to provide treatments for unfulfilled medical requirements, because these materials are frequently biocompatible. Thus, this review focuses on the bacterial pathway for polymer biosynthesis and its potential role in causing pathogenesis, as well as how it can have diverse industrial, technical, and medical applications.

## 2. Types of Bacterial Biopolymers

### 2.1. Polysaccharides

**Figure 1:**
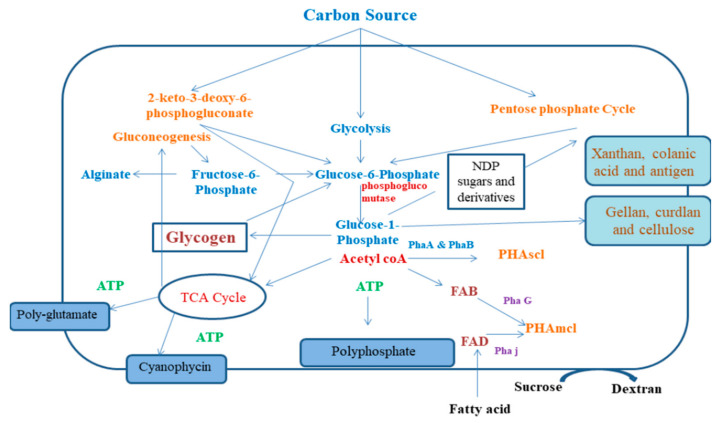
Mechanism of bacterial polymer synthesis pathway. FAB: fatty acid de novo biosynthesis; FAD: fatty acid β-oxidation; KDPG: 2-keto-3-deoxy-6-phosphogluconate pathway; NDP: nucleoside 5ʹ-diphosphate; P: phosphate; PGI: phosphoglucoisomerase; PGM: phosphoglucomutase; Pha: polyhydroxyalkanoate synthesis enzyme; TCA cycle: tricarboxylic acid cycle.

Polysaccharides are mainly divided into two categories, namely heteropolymers and homopolymers. These polysaccharides can be uncharged or charged, repeating or non-repeating, and un-branched or branched. A plethora of bacteria can produce polysaccharides and reserve them within their cells, such as glycogen, or they may also secrete polysaccharides, which are attached to the cell surfaces or free exopolysaccharides that help in the matrix formation in biofilms (cellulose and alginate mainly) [7]. On being motile, pathogenic organisms produce various virulence factors and other toxic substances such as exotoxins. On the other hand, while in sessile state, pathogens produce different exopolysaccharides such as cellulose, alginate, and hyaluronate, acting as components of biofilm matrix. This shifting to sessile biofilm state defines the onset of chronic infectious diseases, since the encapsulated or embedded cells are shielded from host immune responses and anti-microbial drugs [11]. For example, alginate present inside the matrix of biofilm in *Pseudomonas aeruginosa* contributes to enhancing the surviving chances of the bacterial cells by shielding them from phagocytosis [1]. Alginates react with divalent cations forming dense hydrogels with increased water-retention capacity [20]. Again, cellulose production accounts for identical advantages to enterococcal pathogens [21]. *Escherichia coli* is known to produce phosphoethanolamine cellulose that can form a mortar-like structure for stabilization of proteinaceous curli fibers. These fibers strongly interconnect the biofilm forming cells, providing resistivity against conditions of high stress [22]. Other pathogens like *Bacillus cereus* G9241 and *Streptococcus pyogenes* consist of a capsule made of hyaluronate, which is a linear heteropolysaccharide with negative charge and a structural homolog of hyaluronate that is found in the connective tissues of humans. Thus, these pathogens can easily escape from being exposed to phagocytosis and opsonization. Serogroup B *Neisseria meningitidis* leads to invasive diseases of meningitis because of the presence of the capsular polysaccharide consisting of sialic acid homopolymers (N-acetylneuraminic acid) having α-2, 8 sialic acid bonds. This polymeric structure of the moieties of polysialic acid resembles the structure of the antigens present in human tissues, and this molecular analogy accounts for reduced immunogenic properties of the capsular polysaccharides, thereby rendering the pathogen non-immunogenic to the host [23]. *Streptococcus pneumoniae* is one of the leading pathogens in causing severe lung diseases and consists of over 100 serotypes producing diverse capsular polysaccharides for evasion of adaptive host immune response [24]. Capsular and secreted polysaccharides are widely used as antigens for producing conjugate vaccines. Designing of serotype-independent vaccines is increasing because of the varying serotypes of *N. meningitidis* and *S. pneumoniae*, which decreases the effectiveness of these vaccines [1]. Glycogen is a water-soluble polymer consisting of α-1, 6 and α-1, 4 glycosidic linkages, and it is the most well-known form of carbon and energy reserve for survival during starvation. In case of intracellular infections, glycogen helps the pathogens like *Salmonella enterica* subsp. *Mycobacterium tuberculosis* and *Chlamydia trachomatis* to survive [25] (Figure 1).

Glycogen is secreted from the capsular polysaccharides that are associated with the surface of the cell and result in the development of the matrix of the biofilm [7]. Alginates that are produced by *P. aeruginosa* are responsible for the protection of the cells from phagocytosis [1]. Phosphoethanolamine cellulose produced by *E. coli* helps in the stabilization of fibers, which in turn form a complex biofilm by interlinkage of these fibres and develop resistance against high shear conditions. *E. coli* produces phosphoethanolamine cellulose, which forms mortar-like structures to stabilize proteinaceous curli fibers. These fibers mediate strong connections between cells in complex biofilms and provide resistance in high-shear conditions. Hyaluronate produced by *Streptococcus pyrogens* and *Bacillus cereus* helps in mimicking hyaluronate being present within the connective tissues, thus protecting them from phagocytosis [26]. It has also been observed that capsular polysaccharide comprising of sialic acid produced by *Neisseria meningitides* resembles the polysialic moieties of human antigen, thereby making it invisible within the host exerting its pathogenicity [23].

Apart from acting as virulence factors, bacterial polysaccharides exhibit distinctive materialistic properties. Synthesis of polysaccharides chemically is tedious, expensive, and restricted to low molecular weight molecules and could be done only for a few types of polysaccharides. Thus, microbial cell factories are essential for manufacturing these polymers. As hydrophilic groups (carboxyl and hydroxyl groups) are present on the polysaccharides, these polysaccharides actually have high water-retention capacity allowing crosslinking (polymer–polymer, drug–polymer, host tissue–polymer, and cellular interactions) and intermolecular interactions. Polysaccharides are able to form porous hydrogels to be used in drug delivery and controlled delivery of anti-cancer drugs [27], enzyme immobilization [28], and therapeutic entrapment of cells, thereby protecting transplanted cells from the host immunity [29] and tissue engineering [30]. Hydrogels that are made from bacterial cellulose can serve as efficient matrices, fiber composites, or hydrogel nanofibrillar scaffold network, having biomedical applications such as wound dressing that can deliver human dermal fibroblasts and epidermal keratinocytes [24]. Cellulose from *Komagataeibacter xylinus* finds application in large scale production of rayon-based fibers to be used as wearable textiles. Hyaluronate produced by non-pyogenic *Streptococcus zooepidemicus* also has wide biomedical applications [31]. Commercialized formulations of hyaluronate forms a gel-like fluid that can be used in injections to treat arthritis pain in knee joints.

Some enzymes naturally alter the materialistic properties of polysaccharides to modify their biological functions. For example, the existence of acetyl groups on chains of polysaccharides remarkably modify structural conformities, thereby affecting chain–chain interactions, water-retention capacity, solubility, molecular weight, and viscoelasticity [32]. Using genetically engineered polysaccharide-modifying enzymes in microbial cell factories or using these enzymes to modify polysaccharides in vitro allows the generation of tailor-made polysaccharides. The fabrication of these materials after modification changes on being blended with other non-polymeric or polymeric components (esterification of stearic acid, crosslinking of citric acid, and plasticizers). Blending accounts for modification of properties like degree of gelation, viscoelasticity, material strength, and porosity. These materials are being used as feedstock components and as bioinks in 3D bioprinting with varied engineering and biomedical applications like drug testing and drug delivery and tissue engineering. 3D cell-loaded scaffolds made from hyaluronate or alginate can be extensively used as artificial extracellular matrix (ECM), providing for a temporary environment for supporting adhesion, invasion, proliferation, and differentiation of different types of cells such as fibroblasts, mesenchymal stem cells, embryonic stem cells, chondrocytes, and osteoblasts [33]. Thus, bacteria can be considered as an important natural source for the secretion of a wide variety of polysaccharides having medicinal uses and potential industrial applications (Table 1).

### 2.2. Polyamides

Bacteria are capable of producing polyaminoacids or polyamides like secreted poly-L-lysine and poly-D-glutamic acid or the intracellularly produced cyanophycin (copolymer of L-arginine and L-asapartic acid), and they can serve as biofilm matrix components or capsular polymer and also storage materials [35]. Just like the polysaccharides of biofilm matrix, polyamide biofilm or capsule is less immunogenic, thereby protecting pathogens like *Bacillus anthracis* from being attacked by the host immune system [33]. Polyamide-based materials are also produced by various non-pathogenic bacteria such as *Bacillus megaterium*, *Bacillus licheniformis*, and many of the cyanobacteria [36]. Polyamides have high charges and may be polycationic or polyanionic. They are considered as non-toxic, biodegradable, and renewable. Metabolic engineering helps in the production of biotechnologically enhanced polyamides. Polyamides potentially substitute chemically produced polymers in industries. For example, poly-D-glutamic acid can be utilized as a flocculant for replacing synthetic flocculants (polyacrylamide or polyaluminium chloride) for treating wastewater [34]. Poly-L-lysine possesses antibacterial properties, since it can disrupt the integrity of membrane, thereby damaging the cross links, and is used in antimicrobial coatings [34].

### 2.3. Polyesters

Polyhydroxyalkanoates (PHA) are bacterially produced bioplastics. They have a linear symmetry and are arranged in hydrophobic spheric inclusion, functioning as a carbon and energy reserving material [10]. Even though many gram-negative and gram-positive bacteria can secrete PHAs, the functions of PHAs and the potential genes involved in PHA-mediated pathogenesis and persistence are still unknown. PHA^−^ mutants of *P. aeruginosa* reveal decreased attachment to surfaces of glass and diminished stress tolerance in presence of biofilms, therefore revealing a significant contribution of PHA in persistence in infections [37]. In one of the plant pathogens *Xanthomonas oryzae* (causes major rice production losses), the regulatory protein called PhaR represses the synthesis of PHA and also suppresses EPS production, thus affecting virulence and phenotypic changes modifying lifestyle of the bacteria [38]. PHAs are reported to be electron sinks, which in anaerobic conditions (terminal electron or oxygen absence) enhance survival [38]. Synthesis and mobilization of PHA are controlled under responses such as environmental stimuli and environmental and nutritional stresses, rendering an advantageous survival.

PHAs are known to be a distinctive bioplastic that can be chemically modified or bioengineered to be used as high-value medical biomaterials such as scaffolds in tissue engineering, sutures, particulate vaccines, and drug carriers or to be used as low-value bioplastic [34]. Synthesis of tailor-made PHAs through physical blending, bioengineering, or chemical modification produced enhanced materialistic properties that met the requirements of medical and industrial usage. Microbial cell factories can be biologically engineered to produce PHA inclusions that can be heavily coated with proteins of functional interest. These functionalized beads of PHA were stable even after being separated from the cell mass of bacteria, indicating high-value performance as immunodiagnostics, vaccines, enzyme carriers, bio separation resins, and tools required in the production of recombinant proteins. Additionally, the functional performance of these protein-coated, non-porous beads of PHA can be tuned further by regulated encapsulation inside porous microspheres of alginate allowing flow-through usage [39,40].

### 2.4. Polyphosphates

Polyphosphate (polyP) is a polymer consisting of condensed phosphates (several inorganic phosphates), which have a high negative charge along with high-energy anhydride bonds. PolyP serves as an energy storing polymer. PolyP biosynthesis results from an evolutionary activity of bacteria, and this helps in providing chemical energy for the various biosynthetic pathways inside bacterial cells and can act as buffers against bases. Additionally, polyPs can act as a metal chelating agent contributing to the formation of channel complexes in order to uptake DNA. PolyPs aid in cell signalling regulation, thus affecting the lifestyle of bacteria, their growth, persistence, viability, virulence, and stress tolerance [41].

Since polyPs can eminently act as an energy-storing material, they have been widely used in industries to provide energy in carrying out enzyme-catalyzed reactions. They are used as biologically active biomaterials in the production of regenerative medicines for repairing cartilages and in the regeneration of bones or can be also used as vehicles for drug-delivery. As polyPs can react with cationic polymers (like hyaluronate and alginate), inorganic cations (like Mg^2+^, Ca^2+^, Zn^2+^, Na^+^, Fe^2+^, and K^+^) or other organic alkaline components (like peptides, polyamines, and amino acids), they can be converted into hydrogels or nanoparticles to be used in biomineralization of bones [42] or for various biomedical uses. Essentially, physical properties like functionality and stability and mechanical durability of polyP-associated complex nanoparticles or hydrogels may change depending upon the class of blended polymeric substances or interacting counterions. This modification furnishes considerable space designing for the generation of a wide variety of materialistic properties for bioinks and smart biomaterials in regenerative medicines. Living organisms, particularly bacteria, are considered as the only source of polyp, since there are no known abiotic polyP minerals found on Earth. Bacteria of the genera *Corynebacterium* and *Mycobacterium* can produce granules of polyP with increased yield and therefore can be used as potential producers for polyP manufacture [43].

### 2.5. Other Bacterial Polymers

Bacteria can even produce various types of biopolymers that can act as proteinaceous components and extracellular DNA. These types of biopolymers are not only responsible for pathogenesis in bacteria, but can be also used for biomaterial production. Extracellular DNA forms when a cell is lysed, releasing the intracellular DNA. In the case of biofilms, a cellular subpopulation lyses to produce extracellular DNA, like for example in stalks of mushroomlike microcolonies formed by *P. aeruginosa* [44]. Because of the high negative charge, extracellular DNA has multiple roles in the adherence and stability of the matrix of biofilm by interaction with cationic polysaccharides (like Pel) and other cations and can also act as a source of nutrients at times of starvation conferring resistivity to antibiotics.

Secreted polypeptides are composed of alternating hydrophobic and hydrophilic residues of amino acids and proteins like pilin, fibrillin, and flagellin. These polypeptides can serve as molecular elementary units to form extracellular self-built structures like pili, fimbiriae, flagella, and functional amyloids such as curli fibers. These self-assembled structures are capable of forming nanotubes or nanofibers, mediating cell adhesion on abiotic and biotic surfaces, developing matrix of biofilm, or providing motility during pathogenesis. Various features like increased ratio of surface area to volume, the explicit arrangement of building blocks of proteins, and polymorphic transformations of these structures are a result of their responses against chemical and physical stimulations, making them a potential component in industrial and biomedical applications. These properties make them important biomaterials and bio templates for the designing of novel nanodevices, nanostructures, and multiple layered lattices to be used in nanomedicines (drug delivery) and bioengineering [45]. The genetic programmability and the simplicity of engineering polypeptides, proteins, and extracellular DNA make them attractive for manufacturing programmable biomaterial-based platforms, which can be hardly manufactured with other types of biopolymers like polyesters and polysaccharides. Moreover, the uncomplicated genetic programmability helped in the development of bioengineered living materials in which the live cells are bioengineered for autonomous self-assembly of all the materials, having tunable and novel properties for different purposes like microbial electrosynthesis, electronic devices for monitoring, biosensors, and bioremediation [46].

## 3. Synthesis and Production of Bacterial Biopolymers

**Figure 2:**
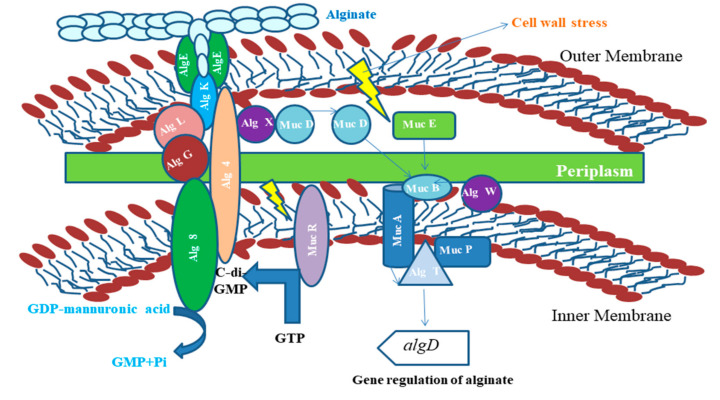
Model for synthesis of alginate and production machinery.

Various biochemical and physical approaches such as functional genomics, genome sequencing, and advanced molecular techniques and tools have evolved and provided more information about the detailed bacterial polymer synthesis and production pathways and processes. Enormous protein and DNA databases in combination with in silico methods provided a perception of the biosynthetic pathways and also the functions and structures of the proteins involved in such pathways. Such advanced techniques have led to the fabrication of cell factories for increased production of biopolymers or tailor-made biopolymers. Recently, approaches developed from synthetic biology using DNA foundries have led to the fabrication of cell factories, allowing precise bioengineering of producer strains [1,47]. Besides the understanding of the biosynthetic pathways and proteins, information about the molecular mechanisms of biosynthesis, modification, and secretion about the innovative tailor-made biopolymers have also come to light. For example, numerous bacterial polysaccharides have been modified enzymatically (acetylated or deactylated, phosphoethanolamine, and epimerized) at the level of polymer synthesis, and such modifications can alter the materialistic properties like gel-forming capability and visco-elasticity [48]. Genes that encode enzymes to be used for polymerization and alterations of polysaccharides are often co-clustered in a main single operon. Specific functional promoters regulate these operons and the transcription process of the whole biosynthetic cluster of genes (Figure 2).

### 3.1. PHA Production

Bacterial strains actively taking part in the accumulation of PHA are used in large-scale industries for production and cost reduction of biopolymers, since they are able to convert waste products into high-value intracellular and extracellular by-products (EPS and PHAs), which can be used in various biochemical processes.

PHAs are considered as intracellular energy and carbon storage materials, and EPS is an extracellular biosurfactant that protects the bacterial cells predation and desiccation. These biosurfactants are used in washing powders and softeners of fabric. For example, 16s rRNA analyses of the PHA-producing isolates of bacteria showed that *Pseudomonas* sp. strain-P(16) is a potential producer of PHA from low cost carbon sources like dates, rice bran, and soy molasses. The productivity of PHA from dates, rice bran, and soy molasses was found to be 82.6%, 90.9%, and 91.6% [49].

Biosurfactants are defined as amphipathic molecules having non-polar and polar heads, and they are produced by bacteria of the genera *Arthrobacter*, *Acinetobacter*, *Pseudomonas*, *Bacillus*, *Enetrobacter*, and *Rhodococcus* [50]. Biosurfactants are composed of a diversity of structures, as their biosynthesis is dependent on the type of carbon sources. They can be produced from different types of sources such as lipids, sugars, alkanes, and other waste products. The major feature of biosurfactant is its capacity to decrease interfacial and surface tension, producing microemulsions.

EPS is a combination of high molecular weight polymers supplying carbon sources under resource limited condition. Wang and Wu (2007) described the EPS biosynthesis from *Ralstonia eutropha*, where they found that EPS production was closely interlinked with the growth of the cells [51]. Thus, they conducted the experiment with different glucose concentrations for evaluation of their influences on EPS production.

Out of all the known EPSs, alginate is essentially important for applications in food industries. It is used in restructured foods, frozen custards, cake mixtures, and cream and beer manufacture. Alginate consists of varying amounts of C-5 epimer α-L-glucuronic acid and β-D-mannuronic acid joined through β-1,4 glycosidic bonds. During alginate extraction from the harvested product, these uronic acids get converted to salts of glucuronate and mannuronate via a neutralization step. The distribution, length, and proportion of these blocks influence the physical and chemical properties of alginate. Commercial alginates are extracted from bacteria such as *P. aeruginosa*, *Azotobacter vinelandii*, and *P. mendocina* [52]. Simultaneous alginate and PHA production from *P. mendocina* by using glucose as the carbon source was studied and performed by Guo et al., 2011 [53].

Simultaneous EPS and PHB production by *Azotobacter chroococcum* and evaluation of the effects of ammonium addition with fructose, glucose, and sucrose was studied by Quagliano and Miyazaki, 1999 [54]. This organism was grown aerobically in shake flasks containing the culture media along with ammonium sulphate at 30 °C for 72 h at 220 rpm. Sucrose was proved to be a better carbon source than glucose with higher production of PHB, whereas glucose was better source for production of EPS.

Therefore, bacteria such as *R. eutropha*, *P. aeruginosa*, *P. mendocina*, *A. chroococcum*, and *A. beijerinckii* can simultaneously produce PHAs and other biosurfactants by utilization of the same organic substrate. Nevertheless, the technological performance of the bacteria during coupled biosurfactant and PHA production leads to a decreased PHA production. Particularly, the optimum operating conditions for biosurfactant and PHA production are actually different [50].

Various fermentative strategies [55] and downstream processing techniques for production and purification of EPS and PHAs [56] have also been described. To make the biopolymers cost-effective, they are being widely produced using various industrial by-products, thereby solving the problem of industrial waste disposal after they are generated. Solid state fermentation (SSF) is a cutting edge biotechnological approach for high fermentation productivity and high concentration of product. For example, *Bacillus subtilis* natto is a widely used GRAS (generally recognized as safe) organism producing levan [57]. Levan can be produced by this bacterial strain through SSF by use of a rapeseed meal as the sole substrate. Levan purification was done by low-pressure liquid chromatography utilizing various types of column fillers. These production strategies can be effectively used to design cell factories for biopolymer production to be used in cosmetics, foods, agriculture, healthcare and medical industries, oil recovery, mining, packaging, textile and printing industries, pharmaceuticals, packaging, and wastewater treatment [58,59].

Most of the design of the cell factories depends on target gene manipulation in the biosynthetic pathways. It is reported that a novel synthetic approach called FENIX has been designed to post-translationally regulate levels of protein [60]. FENIX allows independent regulation of the steady-state protein level and induction of target protein accumulation. The FENIX device is made of a constitutive and proteasome-associated degradation of the desired polypeptide by tagging a hybrid, short, and synthetic NIa/ SsrA sequence of amino acid on the C-terminal domain. Production of proteins is induced through an orthogonal inducer (3-methylbenzoate) addition in the culture media. This system was standardized inside *E. coli* by two tagged fluorescent proteins (mCherry and GFP), and this system was further utilized to totally uncouple accumulation of PHB from growth of the bacterial cells. Through tagging of Pha (3-ketoacyl-CoA thiolase, first step on the pathway), a variable metabolic switch at the node of acetyl-coenzyme A was so designed that the metabolic precursor can be efficiently redirected to form PHB when the system gets activated. The biologically engineered *E. coli* strain attained an excessively high PHB accumulation specific rate of 0.4 h^−1^ along with 72% (*w/w*) polymer content within glucose cultures under a growth-independent system. Therefore, FENIX allows dynamical metabolic flux control in cell factories of bacteria by initiating synthetic post-translational switches in the desired pathway.

An artificial neural network (ANN) model in combination with the algorithm of particle swarm optimization (PSO) was utilized in order to optimize process variables during enhanced production of lipopeptides by aquatic *Bacillus megaterium*, which can use food waste [61,62,63,64]. In the case of non-linear ANN model, pH, temperature, aeration, and agitation were considered as input variables, and concentration of the lipopeptide as the output variable. On applying PSO to the ANN model, optimum values for the process parameters were taken as: temperature = 33.3 °C, pH = 6.7, aeration rate = 128 l/h, and agitation rate = 458 rpm. Under optimal conditions, lipopeptide production was significantly enhanced from the wastes by almost 46% (*w/v*), thereby reducing the operational cost by 20 times in comparison to the traditional synthetic medium. Therefore, the novelty of this approach lies in the fact that the combined ANN–PSO optimization model can enhance the yield of fermentative products such as lipopeptide-based biosurfactants from wastes (Figure 2).

### 3.2. Bioplastic Production

Bioplastics are considered as thermoplastic compounds that are easily biodegradable. They can be produced from the most natural and renewable sources. They are optically active, highly crystalline, and have piezoelectric features. They are mostly linear biopolymers such as hydroxybutyric acid and hydroxylvaleric acid. Such biopolymers are usually produced by the bacterial strain *Ralstonia eutropha* using propionic acid and glucose as substrates. However, wild type *R. eutrophus* utilizes fructose instead of glucose. Therefore, strains capable of utilizing glucose need to be developed for commercial uses. Bioplastic producing bacteria are grown on a large scale with high cell densities inside stirred tank fermenters by using sucrose, glucose, or molasses as carbon sources. Fed-batch systems are usually preferred over continuous systems, and they give a yield of 70–80% bioplastic from *R. eutropha* using medium containing mineral salts along with glucose and propionate as sole carbon sources [12,65,66]. Cells are harvested through flocculation or centrifugation, then recovering the bioplastics using hypochlorite and surfactants for lysing the cells and releasing the intracellular products. Processes of floatation or solvent extraction processes can be also used for bioplastic separation from intracellular products followed by lyophilization or spray drying of the cell mass. Maximum efficiency of bioplastic production can be achieved by cell pre-treatment with methylene chloride or chloroform. The composition and yield of the final product however, depend on the chosen substrate regime.

These bioplastics are today used in the manufacturing of biodegradable plastic bottles, golf balls, coatings impermeable to water, biodegradable packaging, temporary pegs and plates, repairing of bone injuries, and disposable razors.

### 3.3. Xanthan Production

Xanthan is one of the most important industrial biopolymers produced by the gram negative phytopathogenic bacteria *Xanthomonas campestris*. Xanthan is an extracellular polysaccharide composed of repeating units of polymerized pentasaccharide that are assembled together by step-by-step addition of glucose, glucose-1-phosphate, glucuronic acid, and mannose, over a polyprenol carrier of phosphate via a set of enzymatic reactions, in which multiple monosaccharide-specific glycosyltransferases, ketal pyruvate transferase (from nucleotidyl precursor derivatives of GDP and UDP), and acetyltransferases are involved [67]. Xanthan gum is the first industrially used bacterial biopolymer. It is widely used because of its good rheological properties. A high xanthan productivity was obtained by making use of lactose-utilizing *X. campestris*, strain C7 L, growing over a whey media [68]. A two-step fermentation process by combination of filtered whey (0.18% protein content) and unfiltered whey (0.35% protein content) was carried out, where the first stage utilized the unfiltered whey, showing a xanthan productivity of 12 g/L (0.40 g/L/h) with a yield of 45% followed by the second stage, where the addition of filtered whey increased the total xanthan concentration to 28 g/L (0.58 g/L/h) with a yield of 75%. Final viscosity of the broth was found to be 18,000 cP, and the polymer thus produced in this combined system showed thixotropic and pseudoplastic behavior. A relatively lower yield was achieved by using other strains of *Xanthomonas* such as *X. manihotis* and *X. mangiferaeindicae* [68]. Highest productivity of xanthan was (25 g/L; 0.35 g/L/h) obtained by using these two strains when whey from mozzarella cheese was used as the main carbon source in a shake flask culture for 72 h.

However, cultivating *X. campestris* strain ATCC 13951 within shake flasks by using deproteinized cheese whey (CW) as the main carbon source produced a relatively low concentration of xanthan (20.3 g/L; 0.28 g/L/h) [67]. Experiments of optimization were done in shake flasks using *X. pelargonii* strain PTCC 1474 and *X. campestris* strain PTCC 1473 in a culture medium containing deproteinized CW, magnesium sulphate, and phosphorous dihydrogen sulphate [69]. *X. pelargonii* PTCC 1474 showed a relatively lower xanthan production (12.28 g/L; 0.26 g/L/h) in comparison with *X. campestris* PTCC 1473, which showed a xanthan production of 16.65 g/L and productivity of 0.35 g/L/h. Thus, a relatively good yield was obtained with deproteinized CW for hydrolysing equimolar mixture of galactose and glucose.

### 3.4. Gellan Production

Gellan is a polyfunctional gelling agent produced by a gram negative non-pathogenic bacteria *Sphingomonas paucimobilis* strain ATCC 31461. Gellan biosyhthesis is a growth-associated process. The biosynthetic pathway for gellan production is quite complicated, where sugar precursors (UDP-glucuronate, UDP-glucose, and dTDP-rhamnose) are synthesized followed by the repeat unit formation through sequential addition of sugar donors to an active lipid carrier via glycosyltransferases and then gellan polymerization and finally export. This multistep procedure is carried out by a set of enzymes encoded by a cluster of 22 genes [70]. *S. paucimobilis* ATCC 31461 used 2% lactose as the main carbon source (peptone and yeast extract as nitrogen sources) in shake flasks with 180 rpm produced gellan of concentration 16 g/L (0.33 g/L/h) [71].

Another method for gellan production is by using a newer strain called *S. azotofigens* strain GL-1 [72,73]. This bacterial strain was cultivated inside shake flasks at 30 °C and pH 6.5 by utilization of CW medium or molasses. The CW-based medium produced a maximum gellan concentration of 33.75 g/L (0.70 g/L/h). This so obtained gellan possessed a lower glucuronate and higher rhamnose and glycerate content altogether as compared with gellan produced from molasses containing medium.

### 3.5. Hyaluronate Production

Hyaluronic acid (HA) is an anionic linear polymer of repeating units of disaccharides consisting of N-acetyl-D-glucosamine and D-glucuronic acid produced by bacteria such as *Pasteurella multocida* and *Streptococcus* sp. HA is found in tissues of higher organisms and it is a polymer of biomedical importance. The precursors involved in HA synthesis are UDP-N-acetyl-glucosamine and UDP-D glucuronate, and the reaction is catalyzed by the enzyme hyaluronan synthase (Has A) [10]. *Streptococcus zooepidemicus* strain ATCC 25346 after cultivation in medium containing CW or concentrated whey protein produced a moderate HA concentration of 2.14 g/L (0.20 g/L/h) [67].

Another bacterial strain called *S. thermophilus* strain NCIM 2904 can also produce HA by growing on medium containing whey protein hydrolysate (WPH) and whey permeate (WP) supplied with mineral salts [74,75]. This producer strain produced a low concentration of HA (0.343 g/L; 0.014 g/L/h).

### 3.6. Cellulose Production by Bacteria

Bacterial cellulose (BC) is a homopolysaccharide comprising only D-glucose polymers linked via β-1,4 glycosidic bonds formed from precursor UDP-D-glucose via membrane embedded enzyme glycosyl transferase (cellulose synthase BcsA). BC exhibits unique mechanical and functional features, and therefore it is widely used in biomedical applications such as synthesis of biocomposites. *Komagataeibacter xylinus* is widely known for cellulose production. Studies have shown that ethanol has a positive effect on the yield of cellulose by *Komagataeibacter xylinus*, as ethanol provides an alternate source of energy to synthesize cellulose through glucose metabolism. Ethanol helps in the induction of genes that are associated with UDP-glucose formation and represses the genes that are associated with the mechanism of glycolysis [76,77]. BC production is highly expensive, since it is produced either by chemical processes or enzymatically. BC cannot be produced from bacteria that can grow on low cost substrates such as CW or CW permeate. *K. sucrofermentans* strain DSM 15973 produced BC with a very low productivity of 0.0047 g/L/h [78].

Another producer strain called *Gluconacetobacter xylinus* PTCC 1734 growing on a CW hydrolysate medium for 14 days, produced a comparatively higher BC concentration of 3.5 g/L [79]. Moreover, the bacterial strain *K. rhaeticus* P1463 produced a higher concentration of BC (6.55 g/L) by growing on CW hydrolysate medium for 14 days [67,80].

BC finds its application as a wound dressing material for the treatment of chronic wounds, although it does not possess any of intrinsic antimicrobial properties [81]. It has been further observed that the biofilm formation can be inhibited by saturating BC with antimicrobial agents, proving its ability to inhibit the biofilm formation by *P. aeruginosa* [81].

### 3.7. Levan and Dextran Production

Levan is formed by D-fructosyl unit polymerization linked by β-2, six linkages within linear chains with β-2, and one bond within branching points, and this polymerization reaction is carried out by the enzyme levansucrase, which is an extracellular fructosyltransferase catalyzing fructose residue transfer from sucrose to levan, thereby releasing the glucose residues. Producer organism of levan is *A. vinelandii* strain D-08, which can grow on medium containing distillery dregs, milk whey permeate, and molasses in shake flasks at 28 °C and 250 rpm [82]. This producer organism produced a levan concentration of 14 g/L (0.58 g/L/h). Another producer organism *Weissella cibaria* strain growing on a medium containing 5% sucrose and 10% lactose produced a levan concentration of 31 g/L [83].

Dextran is a water-soluble extracellular neutral α glucan consisting of linear chains of repeating units of D-glucopyranosyl linked with α-1, six linkages. Dextran is produced from sucrose by dextransucrase that is an extracellular glucosyltransferase catalyzing slow transfer of residues of D-glucopyranosyl from sucrose to dextran, thereby releasing residues of sucrose. Mostly, lactic acid bacteria (LAB), *Leuconostoc mesenteroides*, is an important producer of dextran used for commercial purposes. Cane molasses or sugar beet is usually used as a medium for dextran production. *L. mesenteroides* can grow well on medium containing whey and sucrose for a reasonable amount of time in shake flask culture at 25–27 °C and 150 rpm [84], producing dextran of concentration 7.23 g/L (0.52 g/L/h).

Column experiments using fine sand and *L. mesenteroides* to produce insoluble biopolymers were monitored for permeability changes through P and S wave responses. It was found that the bacterial biopolymer reduced the permeability by greater than one order of magnitude, involving approximately 10% of porous volume after growing for 38 days [85]. This reduction was due to internal complex structures of the biopolymers, which get accumulated in the throats of the pores. S wave velocity (V_S_) was reported to be greater than 50% when the biopolymer was accumulated, causing a significant stiffening effect on the shear modulus of unconsolidated matrix sediment during confining and low stress conditions. On replacing pore water with insoluble bacterial biopolymer, it was observed that there were minimal modifications in P wave velocity (V_P_) because of the low elastic modulus of the insoluble biopolymer. Upon analyzing the spectral ratio, it was observed that the P wave attenuation increased by 50–80% in both sub-ultrasonic and ultrasonic ranges of frequency, while S wave attenuation increased by almost 50–60%.

## 4. Enhancement in Biopolymer Production

### 4.1. Enhancement of Biopolymer Production by Alteration in Synthesis Mechanism

**Figure 3:**
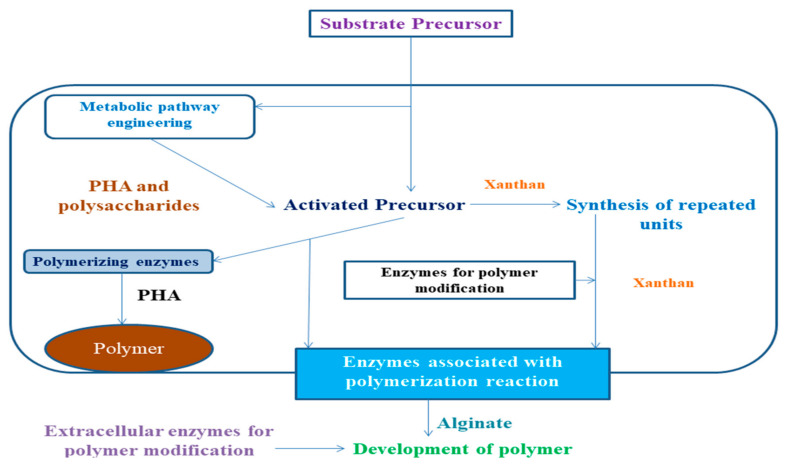
Modification of biopolymer properties by changing the synthesis mechanism.

Sequencing of genome, cloning, functional genomics, and characterization of genes responsible for biosynthesis has immense importance in regulating the biosynthetic pathways taking place within the organisms helping in the discovery of new biopolymers having immense commercial attributes [86]. This also helps in the mechanism of reconstructing and engineering the pathways so better biopolymers can be prepared (Figure 3). Metabolic engineering also plays an important role in exploiting the biosynthesis pathways within the bacterial specie, s resulting in the development of new polymers that include polythioesters and lactate-based-polymers within the recombinant *E. coli* [87].

### 4.2. Enhancement in Biopolymer Production by the Use of Artificial Neural Network (ANN) and Machine Learning

ANN is a computational approach in optimizing a process or process parameters to receive maximum or desired output out of a wet lab experiment [88,89]. ANN is widely used in optimization of enhanced biopolymer production. Studies showed that the production of hyaluronic acid often termed hyaluron, which is a potent biopolymer, can be optimized by the use of ANN [90]. It was further observed that ANN can be implemented in optimizing the parameters for the development of nanofibers with the help of chitosan and poly vinyl alcohol [91].

### 4.3. Enhancement in Biopolymer Production by the Use of Genetic Engineering Technique

The two most important types of bacterial biopolymer are cellulase and alginate, which are found to have numerous industrial applications. It has been observed that the market value of these two polymers is 22 billion USD per year and is projected to rise to 50 billion USD by 2025 [92]. Although at present condition only plants and algae are used for the production of these polymers, various bacterial species have shown their ability of the production of BC and alginate [93,94]. The high demand of these polymeric substances for various commercial purposes, enhancement in their production is warranted through the employment of various newly isolated bacterial species and optimization of their growth conditions [95]. Studies have further shown that the composition of culture does not have its direct impact on the yield of production but determines the morphology of the biomaterials along with their physical properties [96]. A study performed by Chen and co-workers showed that the productivity and quality of BC by Komagataeibacter xylinus ATCC 23770 was drastically enhanced by the utilization of carbon sources obtained from various plant biomass. Supplementation of bacterial growth medium with maltose and glucose resulted in a higher degree of polymerization, increased productivity, and enhanced thermal stability of the polymer, since the added saccharides helped in mimicking the lignocellulose hydrolysate that in turn led to overall enhancement in the production of the biopolymer [97].

The enhancement in the production of bacterial biopolymer can also be achieved by the selective regulation of genes responsible for biopolymer production [98]. Thus, production could be enhanced by using synthetic biology technique that not only can produce improved quality of bacterial biopolymer with desired physical and chemical properties, but also help in constructing bacterial cells possessing greater yields [99]. Study showed that P. aeruginosa PAO1 was modeled for enhancing the production of alginate [100]. production of alginate is regulated by a regulatory circuit MucE being present on the protein associated with the outer membrane. Thus, upregulation of this circuit results in the enhancement in the production of alginate [101].

The adoption of approaches like metabolic and genetic engineering resulted in the enhancement in the production of biopolymers and novel strategy like CRISPR-Cas9, which can be a potential technique to enhance the biopolymer production through specific gene-editing and redirecting the carbon fluxes [99].

Another type of biopolymer Poly-γ-glutamic acid (γ-PGA) showed varied applications depending upon its varied molecular weights. The enhancement in the production of Poly-γ-glutamic acid (γ-PGA) can be achieved by regulation of γ-PGA depolymerase (PgdS) within Bacillus licheniformis.This can be achieved by engineering its promoter and signal peptide, thereby producing poly-γ-glutamic acid of varied molecular weight [73].

The toxic hydrocarbons can also be used as a potent precursor for biopolymer synthesis [102], which will help in production of commercially important compounds with effective bioremediation [92].

## 5. Applications of Bacterial Biopolymers

**Figure 4:**
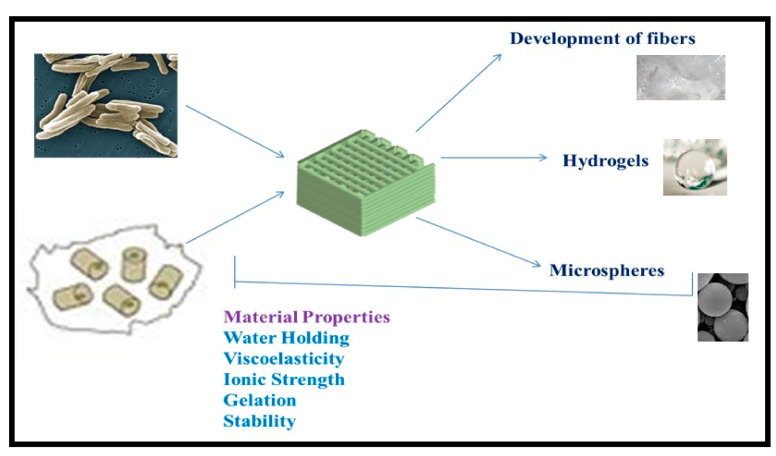
Application of biopolymers from bacterial cells.

Bacterial biopolymers not only lead to pathogenesis, but also provide a plethora of industrial as well as biomedical applications. Due to their biocompatibility and biodegradability, they can be used to enhance the performances and functions of other bioactive molecules in a particular product. These biopolymers can be also modified and altered in various ways to meet biomedical and industrial demands (Figure 4).

### 5.1. Biomedical Applications of Bacterial Biopolymers

Over the recent years, biopolymeric materials have raised a great deal of curiosity for applying them in the biomedical field like medical device fabrication, pharmaceutical carriers, and tissue engineering applications [74]. For example, electrospun poly lactic co-glycolic acid (PLGA) scaffolds are extensively used in drug delivery procedures and tissue engineering.

#### 5.1.1. Applications of bacterial cellulose

Bacterial cellulose along with its derivatives is environment friendly. The fields in which cellulose is widely used are listed below.

- Devices for targeted drug delivery: Within solid tablets, cellulose ether leads to a swelling-controlled drug release, since the tablet itself comes in contact with the physiological fluids. This cellulose ether over the surfaces of the tablets starts swelling, thereby forming a chain-like entrapment or a physical hydrogel. As the swelling starts expanding and penetrating from the surfaces to the glass-like core of the tablets, the drug readily gets dissolved in water, diffusing out through the network of polymers [103].
- Scaffolds used in regenerative medicines: Since BC is extensively biocompatible and has excellent mechanical features, so cellulose as well as its derivatives can be widely used as biomaterials for designing and fabrication of scaffolds used in tissue engineering [104].
- Dressings of wounds: BC has a unique property for wound healing because of its high water holding capacity and purity [105].

#### 5.1.2. Applications of Alginates

Alginates are hydrocolloids used for matrix supporting or used as a system to deliver drugs for tissue repairing and regeneration [106]. Due to its non-antigenicity, biodegradability, biocompatibility, and chelating capacities, alginate is extensively used in various biomedical fields such as drug delivery, tissue engineering, and also in a few formulations to prevent acid reflux. This polymer has been approved by the U.S. Food and Drug Administration (FDA), making alginate an important biomaterial to have been applied in the production of regenerative medicines and nutritional supplements [107]. Apart from these, alginates are even used in the making of impressions in dental hospitals, since it is easy to handle [108].

- Dressings of wounds: A mixture of calcium alginate with sodium serves as an effective remedy for alginate dressing for the closure of wounds and haemostasis. They help in providing a moist condition at the site of wound achieving haemostasis and absorbing exudates [109]. They also aid in reduction of wound pain, decrease the biological burden of the wounded site, and absorb proteinases for reducing the odour [110]. Alginate-associated wound dressings include strategies such as electrospun mats, hydrogels, and sponges, offering numerous advantages, like gel-forming capability on absorbing wound exudates leading to haemostasis [111].
- Drug delivery system: Alginate potentially acts as a carrier for immobilization and encapsulation of drugs due to its biodegradability and biocompatibility [106].

#### 5.1.3. Applications of Xanthan Gum

Xanthan gums are highly viscous and exhibit pseudoplastic nature and shear thinning features; thus, it can be said that the viscosity of xanthan decreases with increased shear rate. Xanthan gum is immensely used in cosmetics and toothpastes [112]. It can be easily squeezed out from the tubes. It even makes sure that the toothpaste remains stable on top of the brush. The shear-thinning features also enhance dispersal of dirt from the teeth, thereby rinsing the teeth. Condensed toothpaste made from xanthan gum has a shiny and bright cord showing a short flow characteristic. In suspensions or emulsions for pharmaceutical applications, xanthan gum helps in the prevention of detachment of insoluble components [112].

#### 5.1.4. Applications of Hyaluronic Acid

Hyaluronic acid (HA) has also been approved for use in injections by the U.S. Food and Drug Administration (FDA). It is a mucoadhesive, biodegradable, and biocompatible polysaccharide having a negative charge. Hyaluronan was first used as a substitution/ replacement for vitreous humor during surgeries of the eyes during the late 1950s. Polymers of HA are usually applied in gel preparations for drug delivery in eyes or other optical cavities. Corneal shields made from HA are known to demonstrate prolonged delivery of steroids along with a quintessential dosage profile smoothing [113]. Apart from these, there are various other uses of HA as observed by Fakhari and Barkland, 2013 [114], are described below:

- Epithelial regeneration
- Extracellular regeneration leading to wound healing
- Agent of viscosity in pulmonary diseases to achieve alveolar patency
- Topical medicinal treatment for treating Sjogren’s syndrome (dry eye syndrome) [115]
- Regenerative medicinal filler to cure cutaneous lines and wrinkles
- Commercial formulations to be used in intra-articular injections

Viscosupplementation with derivatives of HA aids in improving physiological conditions within an osteoarthritic joint by supplementation of lubrication and shock absorption properties of synovial fluid in osteoarthritis. The main objective of using viscosupplementation is restoration of the shielding viscoelasticity of synovial hyaluronan, pain reduction and mobility improvement [116]. Esterified HA is used in prevention of bacterial adherence on dental implants, catheters, and intraocular lenses. HA is considered as a potential matrix/support element in dermal augmentation and regeneration. Due to its hydrate forming ability and expansion capabilities, HA is effectively used in cosmetic surgeries such as augmentation of soft tissues [117].

#### 5.1.5. Applications of PHB

PHB is an environmentally friendly substituent of synthetic or artificial thermoplastics, since it has similar properties to those of the synthetic polymer polypropylene and is entirely biodegradable on being subjected to natural conditions after being disposed of. Thus, substituting nonbiodegradable polymers with eco-friendly and completely biodegradable biopolymers such as PHB will serve the purpose of combating ecological problems resulting from production, usage, and disposal of artificial polymers [118]. The various uses of PHB are given below:

- Scaffolds in tissue engineering: PHB has well known applications in the manufacture of scaffolds used in tissue engineering, because they have ideal materialistic properties like biocompatibility, can very well support growth of the cells, and also help in guiding and organizing cells.
- Growth of cells: PHB acts as a unique biomaterial for supporting the growth of different types of cells such as osteoblasts, fibroblasts, umbilical endothelial vein cells in humans, smooth muscle cells in aorta of rabbits, and chondrocytes derived from cartilage, because PHB can get readily metabolized in the cellular biosynthetic pathway [119].
- Reconstructive surgeries: In vivo and gradual biodegradability of PHB makes it an excellent and potential biopolymer to be used in reconstructive surgeries and to develop cardiovascular products like vascular grafts, pericardial patches, and heart valves [120].
- Controlled system for drug delivery and aids in surgeries: In a controlled drug delivery system, a carrier component that is potentially nonharmful to an organism and has essential mechanical, physical, and biomedical features like biodegradability in biological medium is required. As PHB along with its derivatives match these criteria, so they can be used in controlled drug delivery processes, manufacturing of sutures, surgical pins, swabs, wound healing, bone plates and replacements, orthopaedic applications, and remodeling of cartilage [121].
- Peripheral implants and substitutes: PHB is utilized as a pericardial replacement and blood vessel substituent, and it also serves the purpose of stimulating growth and healing of bones and acts as dental and cardiovascular implants because of its piezoelectric characteristics [122]. Sodian et al., 2000, also found that PHB can be successfully used in the fabrication of porous, biodegradable, three-dimensional heart valve scaffolds [122].
- Various disease treatments: The main product of biodegradation of PHB is 4-hydroxyl butyrate (HB), which is active pharmacologically and is a very promising compound for treating different diseases including narcolepsy, alcohol addiction withdrawal syndrome, cationic and chronic schizophrenia, chronic brain syndrome, atypical psychoses, drug addiction withdrawal, circulatory collapse, Parkinson’s disease, cancer, radiation exposure, and various other neuropharmacological diseases [123]. Units of HB are used to effectively treat narcolepsy, which is a sleeping disorder in humans that is detected during early adulthood causing paralysis, unanticipated sleep attacks, and in few cases, temporary muscle tone loss. HB can even act as a neurotransmitter in the central nervous system (CNS) of mammals, because it has a close chemical and structural homology with gamma-aminobutyric acid (GABA—a regulator of muscle tone), which functions on the receptors for GABA, thereby reducing narcolepsy and regulating the muscle tone [124].

### 5.2. Biopolymer Application in Nanotechnology and Nanoscience

Nanotechnology is the scientific study of nanomaterials dealing with their synthesis, character identifications, and potential applications. Scientists over the recent years are trying to develop more environment-friendly processes to synthesize nanoparticles [74]. Thus, the nanoparticle synthesis procedures have shifted from chemical and physical processes towards using bioprocesses and green chemistry.

Metal nanoparticles, because of their effects from quantum size, exhibit a wide variety of unique properties [125]. On the other hand, most of the nanoparticle synthesis procedures become a threat to the ecosystem. The conventional synthetic methods involve highly toxic reducing agents such as inorganic solvents, N-dimethylformamide, hydrazine, and sodium borohydride. All these chemical compounds have a high reactivity posing a serious ecological and biological risk. Due to the increased focus on elimination/ minimization of hazardous wastes, appropriate sustainable processes have been developed using green chemistry strategies. Biopolymers such as BC, poly lactic acid (PLA), and PHA are widely used as reducing and capping agents to synthesize various nanoparticles as the substituent of different toxic reagents [126].

### 5.3. Biopolymer Applications in Food Industries

Substitution of the oil-based packaging materials with biological containers and films provide an added advantage due to their greener and sustainable image, since they have improved technical features. Biopolymers have been recently used as food coatings, matrices of encapsulation for functional foods, and as packaging materials for foods. These biopolymers give unique remedies for improving the shelf life of the products, thereby reducing the total carbon footprint associated with packaging of foods [127]. In food industries, the biomaterials finds application in three primary sectors: food coating, food packaging, and edible films for encapsulating functional foods. The most commonly used biomaterials in food packaging are some biodegradable bioplastics and polyesters such as PHA and PLA, which can be modified using traditional equipment. These bio-based polymers are also used in numerous multilayer and monolayer applications in the field of food packaging. PLA biopolymer is one of the most potentially used biodegradable biopolymers. This is because it has balanced features and is commercially easily available. Particularly PLA is used in food packaging, because it has an exceptionally good transparency along with high water resistivity. The main challenge posed by these biopolymers is their unimproved thermal and barrier properties and so they cannot act like polyethylene terephthalate (PET). The innate processing difficulty and excessive rigidity have limited the use of biopolymers in traditional devices. The hydrophilic characteristics of most of the bio-based materials also limit their uses as high-value products. On absorbing moisture, these biopolymers become plasticized, thereby reducing the barrier qualities of these biopolymers.

Biopolymers are also used in encapsulation processes. Encapsulation is defined as the process by which sensitive substances are protected from effects of adverse environmental conditions. The term microencapsulation is a well-organized process to wrap solids or liquids or gases inside small capsules, which are capable of releasing their constituents under particular circumstances [128]. These technologies are the most important in pharmaceutical industries. Biopolymers are immensely used as edible coatings and films because of their capability to incorporate diverse types of functional components. Plasticizers like polyethylene glycol (PEG), acetylated monoglycerides, and glycerol are used to alter the mechanical features of the coating or film, thereby making significant modifications in barrier characteristics of the films. The main advantage of coatings includes their utilization as an incorporation vehicle for active and natural ingredients like antimicrobial agents and antioxidants, functional components consisting of vitamins, minerals, and probiotics or enzymes. These bio-based ingredients can be consumed along with the food materials, thereby enhancing their sensory and nutritional qualities and ensuring safety of these products for human consumption. Edible films can be used as aroma or flavor carriers besides limiting the loss of aroma [129].

Lysozyme is one of the most widely used antimicrobial enzymes in packaging substances, because it a naturally found enzyme. Biopolymers like amylase, in combination with plasticizers, can be effectively used in the manufacture of thin films for food materials and packaging purposes [130,131].

### 5.4. Biopolymer Applications in Packaging Industries

Today, the most industrially used food packaging materials are specific biodegradable polyesters, since they can be modified using traditional equipment. Among all the bioplastics, the most widely used biopolymer is PHA and PLA [132]. PLA biopolymer is one of the most biodegradable biopolymers. For using biopolymers as packaging materials to package food products, the most significant factor to be considered is their barrier properties. Biopolymers, which are hydrophilic, generally have a low moisture resistivity causing transmission of water vapour via packaging affecting food quality. This in turn leads to diminished shelf life, increased prices, and causing more generation of waste products. Another way of improving the barrier properties of biopolymers is addition of different nanofillers such as nano clays and metal oxide nanoparticles [133]. Out of all the bioplastics, polyglycolic acid (PGA) has exceptionally good barrier properties.

### 5.5. Mechanical Applications of Biopolymers

#### 5.5.1. Use of Biopolymers for Improving Surface Erosion Resistance

The soft biopolymers produced by the bacteria help in altering the resistance against soil erosion. Ham et al., 2018, used the model bacteria *L. mesenteroides* to produce biopolymers that can increase resistance against soil surface and erosion and critical shear stress [134]. They found that the reason behind improving the soil surface erosion resistance is the enhanced cohesive effects of grain-coating slimes of biopolymer, thereby decreasing seepage flows because of the clogged pores as a result of biopolymer formation.

The main polymers that are involved in this mechanism are mainly xanthan and dextran. Insoluble dextran generated from *L. mesenteroides* has been observed to remarkably decrease permeability of soils by greater than one order of magnitude with just a little quantity of dextran, i.e., lower than 10% saturation of the pores. They also reported that an increased content of clay in sands may result in enhanced resistance against erosion and increased heterogeneous erosion characteristics. Thus, from their study, it was concluded that both biopolymer as well as clay content increase significantly the cohesive forces of soils and reduce their void ratio and permeability.

#### 5.5.2. Biopolymer Applications as Construction Binder

Soil treatment followed by improvement is generally performed in geotechnical engineering sections. Various materials and methods have so far been used for soil stabilization and as cement binders in soil engineering. However, the demand for eco-friendly and sustainable approaches is currently on the rise. This is because cement production causes a huge amount of carbon dioxide to be emitted, so there are required substituents like chemical polymers, geochemical, geosynthetics, bacterial biopolymers, and microbial induction. Chang et al., 2016, demonstrated that bacterial biopolymers have a high capacity in replacing cement as a material for soil treatment with respect to their eco-friendly development and production [135]. Without soil binders (like cement), earthen constructions have reduced strength and poor durability. Therefore, bacterial biopolymers can be potentially used as a binder to strengthen soils. Biopolymers vary widely in molecular structures because of their polymerization processes, thereby enabling biopolymer production with customized plasticity or strength, innate biodegradability, and limited emission of carbon dioxide during their production [136]. Thus, these bacterial biopolymers can be effectively used as a substituent against petrochemical products.

Xanthan gum and gellan gum are the mainly used bacterial polymers for preparing binders. Since xanthan can act as a rheology modifier, it can be used to strengthen soil, notably when clayey particles (hydrogen bonds between clay particles and xanthan gum) are present [136]. Gellan gum can form immensely thickened gel at very low concentrations of 0.05–0.25%. Because of its enhanced stability even at low pH and high temperature, gellan can serve as an excessively durable additive for improvement and stabilization of soil [137]. More studies are still going on to ensure the performances of these bacterial biopolymers in terms of reliability, practical implementation, and durability for applications of in-situ biopolymers in the field of geotechnical engineering.

### 5.6. Biopolymer Applications in Microbial Enhanced Oil Recovery (MEOR)

A biopolymer produced by the thermophilic bacteria *Bacillus licheniformis* and a lipopeptide-based biosurfactant obtained from marine *B. megaterium* were compared to test their efficacies in enhanced recovery of oil [61]. The unrefined biosurfactant produced by acid precipitation reduced interfacial tension between water and lube oil from 18.6 to 1.5 mN/m and decreased the surface tension of water (deionized) from 70.5 to 28.25 mN/m at a concentration of 250 mg/ l. The biosurfactant showed highest emulsification activity (E_24_) of 81.66% against lube oil. Adding calcium ions to the solution of biosurfactant stabilized the lipopeptide micelles. The efficiency of oil recovery in the case of calcium ion conditioned solution of lipopeptide in a sand-packed column was being optimized with the help of ANN modelling in combination with genetic algorithm (GA) approach. Three major factors, namely concentration of lipopeptide, pH of the solution, and concentration of calcium ions, were taken into consideration for carrying out the optimization studies.

With the aim of further improving the recovery efficacy, a soluble biopolymer from *B. licheniformis* served as flooding agent after incubation of the biosurfactant. After ANN–-GA optimization, oil recovery of 45% was attained when the biopolymer as flooding agent was present at a concentration of 3 g/L. On using only water as a flooding agent, there was 29% oil recovery at optimum conditions as predicted by the ANN–-GA optimization strategy. They also tested the various essential features like viscosity, bio-cementing capacity, and pore plugging capacity of the biopolymer. Thus, biosurfactant incubation in combination with biopolymer flooding, under optimum process conditions, showed an oil recovery of 45%. Therefore, it can be concluded from this study that the combined interplay between biopolymer and biosurfactant aided in mobilization and solubilization of oil from the soil.

In another study conducted by Jeong et al., 2019, they examined the MEOR process efficiency on the basis of in-situ selective plugging through production of bacterial biopolymers and then optimization with nutrient injection approach for obtaining highest oil recovery [138]. This experiment was done based on the in situ selective plugging by the production of the bacterial biopolymer called dextran from *L. mesenteroides*. Dextran production and bacterial growth were explained using a stoichiometric equation and kinetic reactions by using batch model simulation. On the basis of the parameters for permeability lowering generated by the sandpack model, the process of MEOR was materialized in a pilot-scale plant, which included an extremely permeable thief zone within a reservoir with low permeability. The basic MEOR process achieved 61.5% enhancement of the recovery factor as compared to that achieved with only waterflooding. The simulations of the parameters indicated that the efficiency of recovery was affected by the quantity and distribution of dextran, proving that the injection approach is critical in controlling distribution of dextran. On incorporation of the results obtained from the sensitivity optimization and analysis, it was concluded that there was an improvement of 36.7% in the efficiency of oil recovery using the optimized process of MEOR as compared to that of the basic process.

## 6. Conclusions and Future Perspectives

Extracellular biopolymers, which are produced by pathogenic bacteria, act as significant virulence factors. Thus, inhibiting their biosynthetic pathways is one of the strategies to treat infections caused by bacterial pathogens. Due to the increased degree of antimicrobial resistivity, newer strategies must be developed in order to combat bacterial infections. More studies on biopolymer synthesis, production, and regulation should be carried out to find improvised and suitable drug developing strategies such as weakening of the bacterial immune system against antimicrobial treatment or host immunity.

Non-pathogenic bacteria also produce numerous biopolymers that can be used for various biotechnological applications in medicines as well as in industries. In spite of achieving great improvements in the designing and fabrication of the cell factories for increased production of natural and tailor-made biopolymers, there still exist some limitations such as the huge cost, validation of safety profile of these biopolymers, etc. [34]. Due to a wide variety of interplaying components’ multi-feedback loops in complicated bio-systems, some reasonable bioengineering strategies for new GRAS-guaranteed microbial cell factories and biopolymers are still not extensively researched. It is necessary to diminish the complicacy via systems biology in order to improve metabolic models for genome-scaling and metabolic modeling of networks, along with computational simulations involving large sets of databases feeding into synthetic biology strategies.

In this review, we have focussed on the understanding of the various biosynthetic pathways and microbial cell-factory designing and fabrication for the production of natural and tailor-made biopolymers, respectively. Additionally, this paper also throws light on the diverse roles of these bacterial biopolymers being utilized for different industrial, geotechnical, and biomedical applications.

## Figures and Tables

**Table 1:** Polysaccharide types of biopolymers.

Type of Polymer	Localization of the Polymer	Primary Structure	Major Component	Precursors	Enzyme for Polemerization and Operon	Producer	Industrial Application	Reference
Polysaccharides								
Hyaluronic acid	Produced extracellularly	β-(1,4) linkage	N-acetyl glucosamine and Glucuronate	UDP–N-acetyl glucosamine and UDP–d-glucuronate	Hyaluron synthase (HasA) *has* operon	Pasteurella Multocida and Streptococcus sp.	Drug delivery, cosmetic, Viscosupplementation, and repair of tissue	[34]
Cellulose	Produced extracellularly	β-(1,4) linkage	D-glucose	UDP-D-glucose	Cellulose synthetase (BcsA) *bcs* operon	Betaproteobacteria, Alphaproteobacteria, Gammanproteobacteria, and Gram-positive bacteria	Wound dressing and in food industry	[34]
K30 antigen	Capsular	β-(1,2) linkage	Glucuronate Mannose,= and galactose	UDP–D-glucuronate UDP–D-galactose, and UDP–D-glucose,	Polysaccharide polymerase (Wzy)	*Escherichia coli*	NA	[34]
Colanic acid	Extracellular	β-(1,4) linkage	Glucuronate, glucose fucose, and galactose	UDP–D-glucose, UDP–D-galactose, GDP–L-fucose, and UDP–D-glucuronate	Colanic acid polymerase (WcaD)	*Shigella* sp., *E. coli*, *Enterobacter* sp., and Salmonella sp	NA	[34]
Gellan	Produced extracellularly	β-(1,3) linkage	Glucuronate, rhamnoseand Glucose	dTDP–rhamnose, UDP–glucuronate, and UDP–glucose	Gellan synthase (Gel G)	*Sphingomonas* sp.	Food additive, culture media additive for encapsulation	[34]
Cudlan	Produced extracellularly	β-(1,3) linkage	Glucose	UDP-glucose	Curdlan synthase (Crd S)	Rhizobium sp., Cellulomonas spp, and Agrobacterium sp.	Food additives	[34]
Glycogen	Produced intracellularly	α-(1,6)-branched and α-(1,4)-linked polymer	Glucose	ADP-glucose	Glycogen synthase (GlgA) *glg* operon	Archea and Bacteria	NA	[34]
Alginate	Produced extracellularly	β-(1,4) linkage	Guluronic acid and Mannuronic acid	GDP–mannuronic acid	Glycosyl transferase (Alg 8) *alg* operon	*Azotobacter* sp. and *Pseudomonas* sp.	Development of biomaterials	[34]
GBS polysaccharides	Capsular		Galactose, Glucose, and N-acetylneuraminic acid; N-acetylglucosamine or rhamnose			*Streptococcus agalactiae*	Currently finding its application in the investigation of vaccines	[34]
Pel	Acetylated -(1,4) linkage	--	N-acetylgalactosamine and N-acetylglucosamine			*P. aeruginosa*		[34]
Psl			L-rhamnose, D-mannose, and D-glucose			*P. aeruginosa*	MEDI3902^d^ (IgG1 mAb) targets Psl	[34]

## Data Availability

The study did not report any data.
